# Anodic Production and Characterization of Biomimetic Oxide Layers on Grade 4 Titanium for Medical Applications

**DOI:** 10.3390/jfb15070180

**Published:** 2024-06-29

**Authors:** Delfina Nowińska, Patrycja Osak, Joanna Maszybrocka, Bożena Łosiewicz

**Affiliations:** Institute of Materials Engineering, Faculty of Science and Technology, University of Silesia in Katowice, 41-500 Chorzow, Poland; delfina.nowinska09@gmail.com (D.N.); patrycja.osak@us.edu.pl (P.O.); joanna.maszybrocka@us.edu.pl (J.M.)

**Keywords:** anodization, biomimetics, oxide layer, porous titanium, drug carriers

## Abstract

Biomaterials are the basis for the development of medicine because they allow safe contact with a living organism. The aim of this work was to produce innovative oxide layers with a microporous structure on the surface of commercially pure titanium Grade 4 (CpTi G4) and to characterize their properties as drug carriers. The anodization of the CpTi G4 subjected to mechanical grinding and electrochemical polishing was carried out in a solution of 1M ethylene glycol with the addition of 40 g of ammonium fluoride at a voltage of 20 V for 2, 18, 24, and 48 h at room temperature. It was found that the longer the anodization time, the greater the number of pores formed on the CpTi G4 surface as revealed using the FE-SEM method, and the greater the surface roughness determined in profilometric tests. As the anodizing time increases, the amount of the drug in the form of gentamicin sulfate incorporated into the resulting pores decreases. The most favorable drug release kinetics profile determined via UV–VIS absorption spectroscopy was found for the CpTi G4 anodized for 2 h.

## 1. Introduction

The combination of medicine and technology is currently one of the most intensively developed fields of materials engineering due to the high and constant demand for newer and better possibilities to improve or extend people’s lives. For this purpose, among other things, numerous studies of various biomaterials are carried out and newer and more efficient techniques for their application are created [1].

Biomaterials are the basis for the development of medicine because they allow safe contact with a living organism. They are used for surgical tools and other medical devices that come into contact with tissues. They are also used for implants. The use of implants, whether biomechanical or aesthetic, is becoming common today and allows the body to continue functioning. Biomaterials are a very specific group of materials that include materials varying significantly in terms of type, chemical composition, and mechanical and physical properties. However, it has a common feature without which a given material could not be referred to as a biomaterial, and this is biocompatibility [1,2,3].

Currently, one of the biomaterials most often used for implants is titanium [3,4,5] and its alloys [6,7,8,9,10]. Titanium, apart from its modulus of elasticity similar to bone, which is the smallest among metal biomaterials, has a number of other advantages [3,4,5,6,7]. First of all, it is characterized by very high resistance to crevices, stress, and general corrosion, even in chloride environments, all thanks to its ability of self-passivation, which results in the formation of a barrier TiO_2_ oxide layer [4,5]. This is an extremely important element when it comes to use in implantology. In addition, titanium also has the highest biotolerance among currently available and used metallic biomaterials. This metal also has an appropriate ratio of yield strength to tensile strength. It also has high mechanical strength, mainly fatigue strength, and this is an important element when it comes to the durability of implants. It has a low density of 4.54 g cm^−3^ and paramagnetic properties [11,12,13].

Among all available varieties of titanium, Grade 4 has the highest mechanical strength. At the same time, it has good plasticity and good impact properties at low temperatures. It can be processed like other classes, cold or hot. It can be welded and cast. It is most often used in the production of aircraft and marine engines, in chemical processing plants, and, above all, in medicine [4,5,11].

The surface of titanium can be modified in various ways, using, for example, titan plasma spray (TPS) [14]; sandblasting, leading to obtaining a resorbable blast media surface (RBM) [4,15]; the deposition of hydroxyapatite (HA) [16,17]; double etching (DE) [18]; Sandblasting Large-grit Acid-etching (SLA) [19], obtaining a hydrophilic surface (SLActive) [20]; oxidation (anodizing) [21,22,23], including plasma electrolytic oxidation (PEO) and also known as micro arc oxidation (MAO) [24]. The creation of differentiated pores on its surface has become very important, thanks to which it connects with the bone tissue much more easily, and due to the elastic modulus being similar to bone, such an implant does not loosen. The porous surface of biomaterials has a biomimetic nature because it imitates the structure of trabecular bone, so it can find numerous applications. One of them is intelligent drug delivery systems (DDSs) [25,26,27]. Thanks to the porous structure resembling a sponge, the medicinal substance can be applied to the pore spaces, surrounded by polymer, and delivered to the living organism. Such a drug carrier releases the drug in a controlled manner, so there is no need to constantly take tablets or maintain appropriate time intervals between doses; in addition, the phenomenon of exceeding the limits of the maximum and minimum amount of the drug substance in the body is eliminated [25,26]. This is an extremely important factor because the drug does not become toxic to the body if the maximum dose is exceeded, nor does it lose its therapeutic power if the minimum dose is exceeded, but it is dosed continuously in an appropriately selected amount. This makes the therapy more effective, safe, and comfortable.

Particularly interesting is the possibility of producing porous oxide layers on titanium surface via anodizing [21,22,23,24,28,29]. Many factors determine the outcome of the oxidation process. Important factors include, among others, the applied voltage, current density, time, chemical composition of the electrolyte, electrolyte temperature, its pH, and blowing the electrolyte with inert gas or mixing [21,22,23,24]. In the case of titanium anodizing, the most frequently used electrolytes are solutions based on orthophosphoric(V) acid with the addition of hydrofluoric acid because this method introduces phosphorus into the layer, which is the main component of bone and contributes to the faster integration of the implant within the bone. The voltage applied to the workpiece can range from 0.5 to 300 V, and the type of oxide layer formed depends on the applied voltage [21,22,23,24,28,29]. A continuous oxide layer, porous oxide layer, or nanotubular oxide layer may be created. The layers created via anodizing can be crystalline or amorphous, stoichiometric or non-stoichiometric, with various morphologies and phase structures.

Anodizing, when performed properly, allows for the creation of an oxide layer with a porous structure [8,9]. This surface is beneficial for implants because it facilitates connections with the bone tissue, which bonds with the pores. The diameter of such pores should be from 300 to 500 μm because this size combines best with bone tissue. Porous layers also have a lower modulus of elasticity, even more similar to bone, which additionally reduces stress and extends the life of such an implant. The thicker the layer, the greater the corrosion resistance and biocompatibility of the metallic material [30,31]. Oxide layers also limit the penetration of ions from the implant surface into the body [8,9].

Oxide layers obtained on the surface of metal implants may have a significant impact on drug delivery systems [25,32,33,34]. The mechanism between the pore characteristics of surface oxide layers and drug loading depends on the way in which drugs are adsorbed, retained, and released from the biomaterials into which they are implemented. Key roles in this multifaceted relationship are played by pore size and distribution, surface chemistry, surface area, porosity, and diffusion, as well as swelling and dissolution [25]. The effectiveness and safety of drug therapies can be increased by designing drug delivery systems with tailored loading capacities and release profiles. The size of the pores on the surface of the oxide layer directly determines the amount of drug that can be loaded. Small pores increase the surface area on which the drug can be adsorbed, thereby increasing the loading capacity. However, when the pores are too small, the penetration of larger drug molecules may be difficult. The pore size distribution may influence the drug release profile. A narrow pore size distribution can result in a more uniform drug release rate, while a wide distribution allows for the tailoring of the release pattern, taking into account the fact that smaller pores release drugs more slowly. The larger the surface area of the oxide layer, the more sites present with which drug molecules can interact. This is a particularly important factor in the case of porous biomaterials in which the ratio of surface area to volume is high. The diffusion of drugs from the biomaterial can be controlled by the porosity of the oxide layer. The tortuosity and connectivity of the pores can slow down diffusion, allowing for a sustained or controlled drug release during the therapeutic period [32].

In the literature, micro- and nanoporous TiO_2_ layers on mechanically polished Ti for the controlled release of tetracycline–hydrochloride have been proposed [33]. Such TiO_2_ layers with a nanotubular structure were produced via MAO and anodic titanium oxide (ATO) treatments in an aqueous solution of 0.15 M calcium acetate monohydrate and 0.02 M glycerol phosphate calcium salt using a pulsed DC field for 3 min. A sol–gel-derived silica xerogel for the controlled release of tetracycline–hydrochloride was loaded into the formed pores. This drug delivery system was characterized by a very high drug loading efficiency in comparison with titanium before surface modification with the drug release for up to 7 days. Recently, to reduce the bacteria-associated infection after implantation, a thermo-sensitive hydrogel composed of biocompatible hydroxypropyl methylcellulose, chitosan, and glycerin was produced on the titanium surface subjected to mechanical polishing and anodizing at 40 V for 1 h in the solution containing ethylene glycol with 0.16 M NH_4_F and 5 vol% deionized water [34]. The author found that this drug delivery system based on the anodic oxide layer with a nanotubular structure as an intelligent drug carrier causes the inhibition of inflammation, thereby inducing macrophage polarization towards the anti-inflammatory M2 phenotype, as well as producing anti-inflammatory cytokines that enhance tissue regeneration.

Inspired by the presented possibilities of the development of modern biomaterials, this work was devoted to developing a new method for the production of porous oxide layers on the surface of commercially pure titanium Grade 4 (CpTi G4) for use in medicine. The proposed research was inspired by intelligent systems for releasing medicinal substances in the body and was to result in the development of a new generation of drug carriers to improve the effectiveness, safety, and convenience of therapy. The first stage was to prepare the surface of titanium samples for testing via mechanical grinding and electrochemical polishing carried out under new conditions in line with the idea of green chemistry. The second stage was the selection of a new anodizing condition, using a solution based on ethylene glycol to create porous oxide layers with a drug-eluting structure on the CpTi G4 surface. In the presented work, the gentamicin sulfate–oxide layer/CpTi G4 drug delivery system is tailored for the first time. The third stage was the characterization of the microstructure, chemical composition, and roughness of the CpTi G4 surface before and after the anodizing process. The fourth stage was the characterization of the release kinetics of gentamicin sulfate from the produced oxide layers as potential drug carriers.

## 2. Materials and Methods

### 2.1. Preparation of CpTi G4 Substrate

Disk-shaped CpTi G4 samples (Bibus Metals, Dąbrowa, Poland) with a diameter of 10 mm and a height of 5 mm were mechanically ground using the metallographic grinding and polishing machine Metkon Forcipol 102 (Metkon Instruments Inc., Bursa, Turkey) on SiC abrasive paper with a grain size of 600# (Buehler Ltd., Lake Bluff, IL, USA). The polished CpTi G4 samples were cleaned for 20 min in the USC 300 TH ultrasonic cleaner (VWR International, Radnor, PA, USA), first in acetone (Avantor Performance Materials Poland S.A., Gliwice, Poland), and then in ultrapure water with a resistivity of 18.2 MΩ cm (Milli-Q^®^ Advantage A10 Water Purification System, Millipore SAS, Molsheim, France).

### 2.2. Electrochemical Oxidation of CpTi G4 Surface

Electrodes were made from CpTi G4 samples by attaching an insulated copper wire to the back wall using chemically resistant epoxy resin (Elecrodag 915 silver paint, TAAB Laboratories Equipment Ltd., Aldermaston, UK). The resin provided electrical conductivity between the CpTi G4 sample and the Cu wire. The next step was to protect the back wall of the samples and their sides with two-component epoxy glue (Distal Classic, Libella Ltd., Warszawa, Poland). The electrodes prepared in this way were electrolytically polished in an acid-free solution containing 200 cm^3^ of ethylene alcohol, 800 cm^3^ of ethylene glycol, 58.5 cm^3^ of sodium chloride, and 10 cm^3^ of ultrapure water. Then, each electrode was immersed in an electrochemical polishing solution poured into a 100 mL beaker and connected as an anode to a Kikusui PWR800H Regulated DC Power Supply (Kikusui Electronics Corporation, Yokohama, Japan). The cathode in the two-electrode system was a mesh made of a platinum–iridium alloy, which had previously been cleaned in HNO_3_ dissolved in ultrapure water in a 1:1 proportion, rinsed thoroughly with ultrapure water, and then air-dried before immersion in the electrochemical polishing solution. Electrochemical polishing was performed at room temperature and the applied constant voltage (U) was 20 V. The duration of the first polishing part (t) was 50 min. Each electrode was then disconnected and rinsed with ultrapure water and then reconnected and electrochemical polishing continued under the same conditions for another 10 min.

Then, the process of the electrochemical oxidation of the CpTi G4 surface was carried out in the same system as in the electrochemical polishing process. A solution of 1M ethylene glycol with the addition of 40 g of ammonium fluoride was used for the electrochemical oxidation process. The anodizing was carried out at a voltage of 20 V and for 2, 18, 24, and 48 h at room temperature. After anodizing, the anode was rinsed with ultrapure water.

### 2.3. Materials Characterization

The CpTi G4 surface before and after electrochemical oxidation was subjected to microstructure tests using a Hitachi HD-2300A field-emission scanning electron microscope (FE-SEM) (Hitachi Ltd., Tokyo, Japan) in a low vacuum of 50 Pa and an accelerating voltage of 15 kV and Hitachi 25 TM4000/TM4000Plus II—Hitachi High Technologies (Hitachi Ltd., Tokyo, Japan) in a low vacuum with an accelerating voltage of 20 kV. Thanks to the high efficiency of the secondary electron detector, it was possible to obtain high-resolution FE-SEM images. A quantitative examination of the surface chemical composition and the surface distribution of elements was also performed using an energy-dispersive spectroscopy (EDS) detector.

The porosity of the oxide layers obtained on the CpTi G4 surface was determined using a computer porosity testing method. SEM images illustrating the surface morphology of the anodic oxide layers were analyzed with the ImageJ program using the JPOR macro based on the thresholding technique [35]. The JPOR plug-in was used as a macro toolkit designed to work with ImageJ to measure porosity from the acquired microscopic images.

The non-destructive testing of the thickness of the obtained porous oxide layers on CpTi G4 was determined using a Coating Thickness Gauge Novotest TP-2020 thickness gauge (Blum—Novotest GmbH, Grünkraut, Germany), equipped with an NF-2 probe dedicated to measuring the thickness of anodized oxide layers on non-ferrous metals [36]. For each tested sample, 10 measurements were carried out in different places on the surface and the result was presented as the average value with standard deviation.

The surface roughness of the obtained oxide layers and the CpTi G4 substrate was measured via the contact profilometry method using a Mitutoyo Surftest SJ-210 profilometer (Mitutoyo Corporation, Kanagawa, Japan). The contact measurement of the geometric structure of the tested surfaces was performed in accordance with the ISO 21920-3:2022-06 standard [37]. The speed of the measuring needle was 0.5 mm s^−1^, and the measurement covered a section of 4 mm. Five measurements were recorded for each sample and their average was presented as the result. Three samples of each type were tested.

### 2.4. The Implementation and Release Kinetics of Gentamicin Sulfate from the Oxidized Surface of CpTi G4

**Figure 1:**
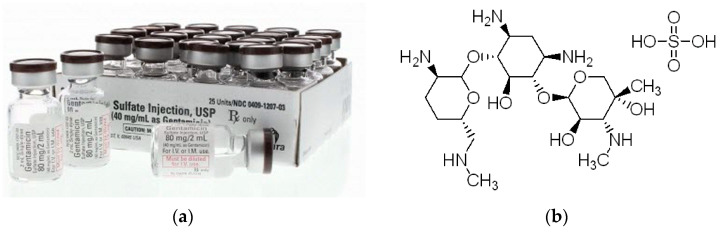
Gentamicin sulfate: (**a**) injection [38]; (**b**) structural formula (C_60_H_125_N_15_O_25_S) [39].

To assess the possibility of using the porous oxide layers obtained on the CpTi G4 surface as drug carriers, the selected drug was implemented in the form of gentamicin sulfate (Sigma-Aldrich, Saint Louis, MI, USA), which is commercially available (Figure 1a) [38]. The structure of gentamicin sulfate is shown in Figure 1b [39]. Gentamicin sulfate is a medicinal substance that belongs to the group of aminoglycoside antibiotics and has a bactericidal effect [38,39]. It is used in clinical practice in the case of infection of both hard tissues, bones, and soft tissues, and prophylactically to prevent postoperative infections. The mechanism of action of gentamicin sulfate is to block the synthesis of bacterial proteins. Its action depends on the drug’s penetration into the bacterial cell, where the ribosomes are located. The selection of gentamicin sulfate as the drug for loading into porous oxide layers, particularly in the context of medical implants or drug delivery systems, was influenced by several criteria and considerations including broad-spectrum antibiotic, high potency, bactericidal action, local delivery, stability, precedent in clinical use, compatibility with materials, and release kinetics [40].

Before drug implementation, the CpTi G4 samples with porous oxide layers were subjected to surface functionalization via heparinization by mixing 40.00 mg mL^−1^ heparin with 19.06 mg mL^−1^ N-(3-Dimethylaminopropyl)-N’-ethylcarbodiimide, hydrochloride (EDAC), and 11.50 mg mL^−1^ N-Hydroxysuccinimide (NHS). This mixture was added to 10 mL of MES buffer (2-(N-morpholine) ethanesulfonic acid) with pH = 4.5(1) and mixed together for 10 min (solution I). A solution consisting of 102.20 mg mL^−1^ of dopamine and 1 mL of MES buffer with a pH of 4.5(1) was added (solution II) to the mixture thus obtained. Each CpTi G4 sample with a porous oxide layer was placed in a separate container with the obtained mixture, tightly secured against spilling, and subjected to a heparinization reaction via mixing with a laboratory shaker at a speed of 40 rpm for 12 h. After this time, the samples were removed from the solution, dried, and then immersed in a solution containing 250 mg of gentamicin sulfate dissolved in 5 mL of ultrapure water. The samples were kept in the drug solution for 48 h at 37 °C.

The release kinetics of gentamicin sulfate from porous oxide layers obtained on the CpTi G4 surface was examined by immersing the samples in 15 mL of phosphate buffer (PBS), the pH of which was 7.4(1) at 37 °C. The release kinetics of the implemented drug substance was examined every 24 h for 48 h, and every hour in the first 3 h. The tests were conducted for three samples of each type, and the average values of gentamicin sulfate release were expressed as percentages. Each time, 1.5 mL of the solution was taken, and the same amount of fresh solution was added. The study was performed using the UV–VIS absorption spectroscopy method (Biochrom WPA Biowave II UV/Visible Spectrophotometer, Cambridge, England). Absorbance values were measured at a wavelength of λ = 275 nm using a quartz measuring cell. Initially, the absorbance value of the drug solution before and after the implementation of the drug was characterized, and then of the sampled solution after drug implementation.

### 2.5. Fourier-Transform Infrared Spectroscopy Measurements

Fourier-transform infrared spectroscopy (FTIR) measurements involved passing an infrared light beam through the tested samples and revealing the amount of energy absorbed at each wavelength. Thanks to this, it was possible to generate transmittance or absorbance spectra, and their analysis made it possible to learn the details of the molecular structure of the tested samples. Infrared radiation covered the range of 400–4000 cm^−1^. Thirty scans were performed. The result of the study was an interferogram transformed into an absorption spectrum. The spectra were taken using the attenuated total reflection–Fourier-transform infrared spectroscopy (ATR-FTIR) method. A Shimadzu IR Prestige-21 FTIR spectrophotometer (Shimadzu, Kyoto, Japan) was used for the study, which was equipped with an ATR reflectance adapter with a diamond. The ATR-FTIR method was used in the research to reveal the structure of the TiO_2_ oxide layer on the CpTi G4 surface before and after anodizing and confirm the presence of gentamicin sulfate incorporated into porous oxide layers on the Ti G4 surface.

## 3. Results and Discussion

### 3.1. Electrochemical Characteristics of the Process of Creating Porous Oxide Layers on the Surface of Cp Ti G4

**Figure 2:**
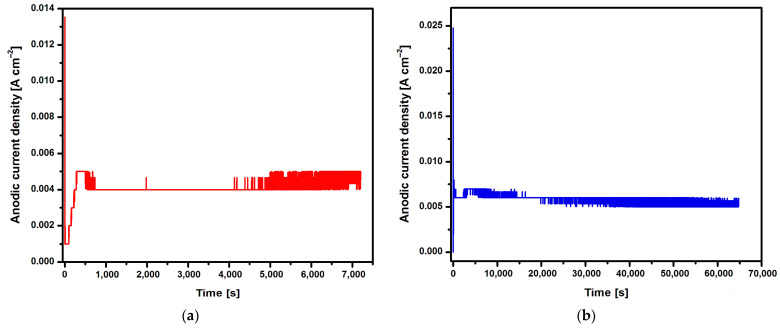

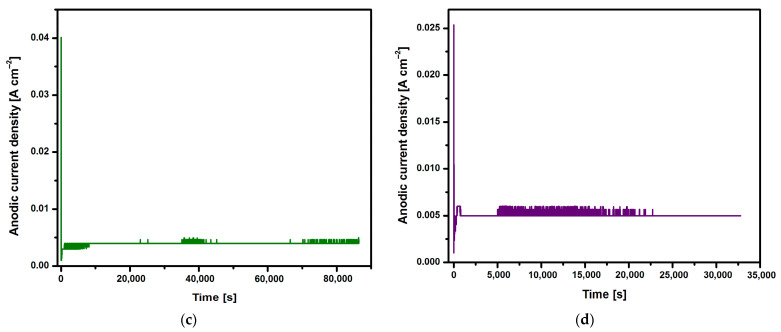
Changes in anodic current density at voltage U = 20 V in the CpTi G4 anodizing process over time for (**a**) 2 h; (**b**) 18 h; (**c**) 24 h; and (**d**) 48 h.

Changes in the anodic current density at a voltage of U = 20 V during 2, 18, 24, and 48 h of the electrochemical oxidation of the Ti G4 surface to produce porous oxide layers are shown in Figure 2a–d, respectively.

Based on the shape of the obtained current–time characteristics for the CpTi G4 under the proposed electrochemical oxidation conditions, one can notice the repeatability of the course of the obtained curves shown in Figure 2a–d. Electrochemical noise is also visible, resulting from the semiconductive properties of the oxide layers formed on the surface of the CpTi G4 substrate. At the beginning part of each curve, there is a sudden drop in the anodic current density from the initial values in the range of 0.014 to 0.040 A cm^−2^ to values close to 0 A cm^−2^ (stage 1), after which the anodic current density increases to a value oscillating around 0.005 A cm^−2^ (stage 2). Then, a plateau is observed, the length of which increases with the anodizing time, which ranges from 2 to 48 h (stage 3). This nature of changes in the anodic current density as a function of time during the electrochemical oxidation of CpTi G4 proves the multi-stage process of creating a porous oxide layer [21,22,23].

The process of the electrochemical formation of porous TiO_2_ layers involved anodizing a metal titanium substrate in an electrolyte solution containing ammonium fluoride dissolved in ethylene glycol. Fluoride ions played a key role in chemically dissolving the oxide layer formed on the titanium surface, which was an essential step in the formation of pores according to the electrochemical reactions described in Equations (1)–(3):

Ti → Ti^4+^ + 4e^−^, (1)

Ti^4+^ + 2OH^−^ → TiO_2_ + H_2_O, (2)

TiO_2_ + 6F^−^ +4H^+^ → [TiF_6_]^2−^ + 2H_2_O. (3)

When a required voltage is applied to the titanium anode, metal atoms at the electrode surface are oxidized to form titanium dioxide. Equation (1) describes the reaction of titanium oxidation, where the metal loses four electrons to form a titanium(IV) ion. The oxidation state of titanium changes from 0 in the elemental form to +4 in the ionic form. Four electrons from the titanium atom are transferred to an oxidizing agent, not explicitly mentioned in the reaction, which gains these electrons, undergoing reduction as part of a redox reaction. The synthesis reaction described by Equation (2) represents the formation of titanium dioxide and water from titanium(IV) ions and hydroxide ions. Then, the fluoride ions originating from the ammonium fluoride in the electrolyte react with the formed TiO_2_ to create a soluble hexafluorotitanate(II) complex, which is then dissolved into the electrolyte (Equation (3)). Water is also a product of this complexation reaction. The ethylene glycol in the electrolyte acts as a solvent and also influences the growth of the pores by affecting the ion mobility and the electric field distribution. The viscosity of ethylene glycol is higher than that of water, which can lead to a slower dissolution rate.

In the first stage, a thin and compact barrier layer is created by thickening the self-passive oxide layer (TiO_2_). In the second stage, the barrier layer is rebuilt at the interface of the oxide layer|electrolyte, and a porous layer is formed. The volume of TiO_2_ formed is larger than the volume of the reacted metal, as a result of which tensile stresses appear in the oxide layer, which leads to cracks in the barrier layer. The emerging cracks promote the formation of pores and the diffusion of electrolytes into them [21,22,23]. The moment at which the anodic current density is stabilized at the level of 0.005 A cm^−2^ corresponds to the beginning of the process of producing an oxide layer with a porous microstructure (Figure 2). In the third stage, only the thickening of the porous layer is observed. The increase in the thickness of the oxide layer occurs through the deepening of the pores formed as a result of two competing processes, namely the formation of a porous oxide film and its dissolution by the electrolyte. It was experimentally found that the thickness of the obtained oxide layer on the CpTi G4 increases with the anodizing time and is 0.67(12) µm after 2 h, 3.17(14) µm after 18 h, 4.24(61) µm after 24 h, and 5.88(79) µm after 48 h.

### 3.2. FE-SEM Characterization of the CpTi G4 Microstructure before and after the Electrochemical Oxidation Process

**Figure 3:**
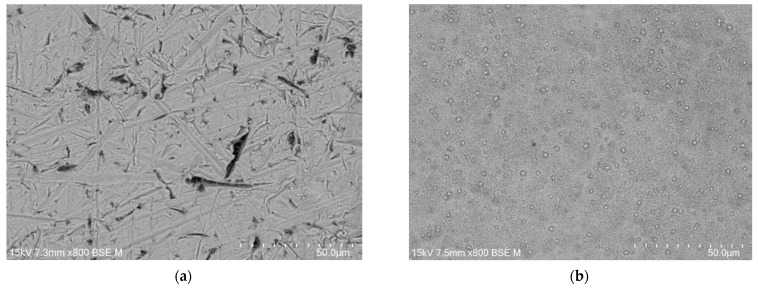
FE-SEM image of the CpTi G4 microstructure: (**a**) after mechanical grinding; (**b**) after electrochemical polishing.

FE-SEM images of the CpTi G4 microstructure after the initial mechanical grinding on #600 grit abrasive paper and electrochemical polishing are shown in Figure 3a,b, respectively.

The microscopic FE-SEM image of the CpT G4 surface after the mechanical grinding shows numerous scratches and micro-cavities remaining after grinding (Figure 3a). One can see that obtaining a mirror-like surface of the CpTi G4 requires subsequent stages of mechanical grinding using abrasive papers of higher gradation and mechanical polishing using polishing pastes or suspensions. The surface morphology of the CpTi G4 is smoothed after electrochemical polishing (Figure 3b). Based on the obtained FE-SEM results, it can be concluded that electrochemical polishing significantly improved the surface quality of the CpTi G4, removing imperfections in the surface microstructure and allowing for the obtainment of a continuous and uniform oxide layer, which can additionally increase the corrosion resistance in a biological environment compared to an ultra-thin self-passive oxide layer [4].

**Figure 4:**
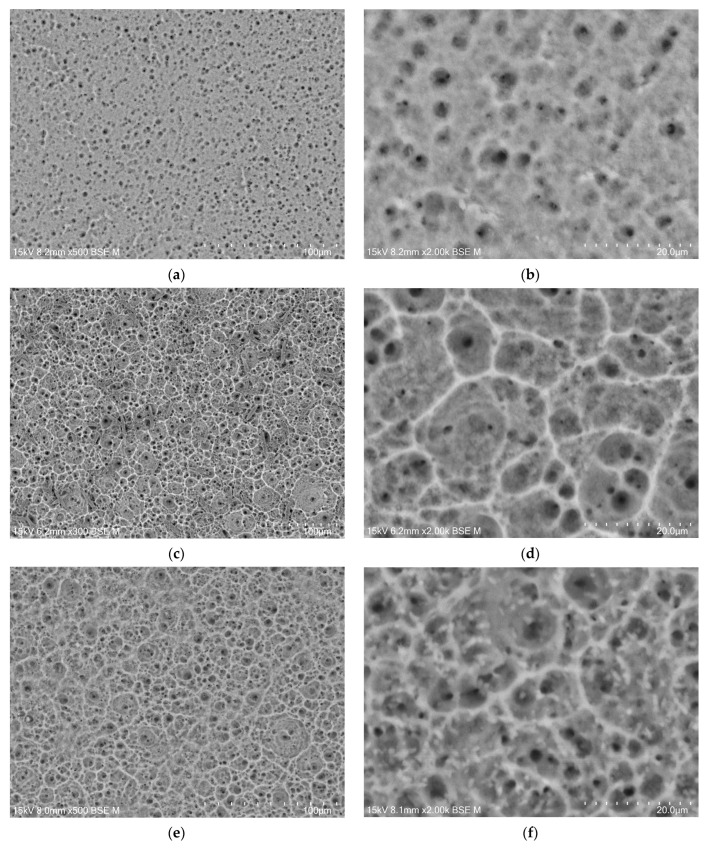

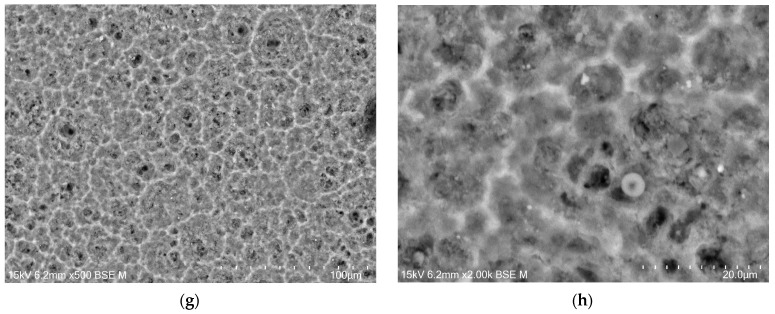
FE-SEM images of the microstructure of a porous oxide layer formed on the CpTi G4 surface in a solution of 1M ethylene glycol with the addition of 40 g of ammonium fluoride at a voltage of U = 20 V at a time of (**a**,**b**) 2 h; (**c**,**d**) 18 h; (**e**,**f**) 24 h; and (**g**,**h**) 48 h.

The microstructure of the oxide layers produced on the CpTi G4 surface at a voltage of 20 V for electrochemical oxidation times of 2, 18, 24, and 48 h is shown in FE-SEM images in Figure 4a–h. In all the obtained microscopic images, the microporous structure of the surface of the oxide layers can be observed.

Based on the obtained FE-SEM images visible in Figure 4a–h, it can be concluded that the longer the electrochemical oxidation time, the more extensive the pore microstructure on the CpTi G4 surface becomes. During electrochemical oxidation lasting 2 h, pores were formed in the form of points distributed on the solid surface. Electrochemical oxidation for 18, 24, and 48 h provided a more developed microstructure, which is characterized by a complex pore structure. It can be observed that smaller pores were formed in larger pores, and the resulting microstructures began to resemble the structure of the spongy (cancellous) substance found in bone [41]. The creation of such a homogeneous and porous microstructure during electrochemical oxidation was possible thanks to the previously prepared CpTi G4 surface, which was electrochemically polished. The size of the micropores on the CpTi G4 surface increases with anodizing time. For the CpTi G4 sample anodized at 20 V for 2 h, it ranges from 1.45(8) µm to 2.45(11) µm (Figure 4a,b). In the case of the CpTi G4 sample anodized at 20 V for 18 h, the micropore size ranges from 3.55(5) µm to 12.03(3) µm (Figure 4c,d). The size of micropores on the surface of the CpTi G4 sample anodized at 20 V for 24 h ranges from 3.44(4) µm to 19.04(8) µm (Figure 4e,f). The CpTi G4 sample anodized at a voltage of 20 V for 48 h is characterized by a micropore size ranging from 5.36(9) µm to 31.67(10) µm (Figure 4g,h).

**Figure 5:**
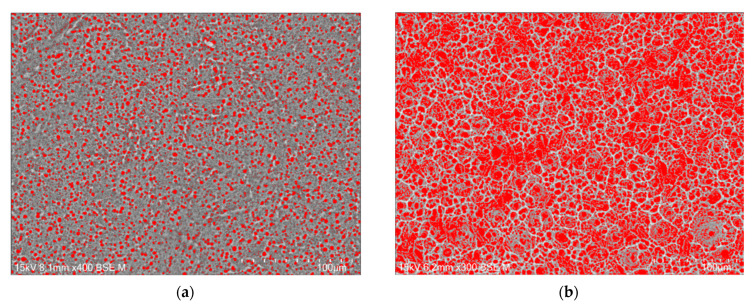

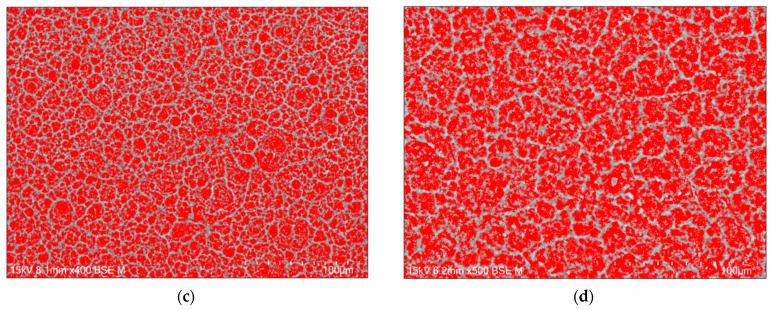
Computer analysis of porosity based on FE-SEM images of the microstructure of a porous oxide layer formed on the CpTi G4 surface in a solution of 1M ethylene glycol with the addition of 40 g of ammonium fluoride at a voltage of U = 20 V at a time of (**a**) 2 h; (**b**) 18 h; (**c**) 24 h; and (**d**) 48 h.

The porosity of the oxide layer on the Cp TiG4 surface obtained after 2 h of anodization is 31(1)% (Figure 5a), after 18 h of anodization 48(4)% (Figure 5b), after 24 h of anodization 54(4)% (Figure 5c), and after 48 h of anodization 57(3)% (Figure 5d).

The observed pores connect and form inside already existing pores, which indicates the formation of trabecular scaffolds with promising osteoconductive properties [42]. The authors of the presented work aimed to obtain a porous structure similar to the spongy substance of human bone, which is characterized by irregular pores. They also determined the porosity of bone tissue, which ranged from 50 to 90%, depending on the condition of the bone tissue of individual people. The obtained porosity results for porous oxide layers on the CpTi G4 surface indicate that under the proposed anodizing conditions, a biomimetic structure can be obtained that will perfectly imitate the trabecular structure of bone tissue under in vivo conditions.

### 3.3. EDS and ATR-FTIR Study

**Figure 6:**
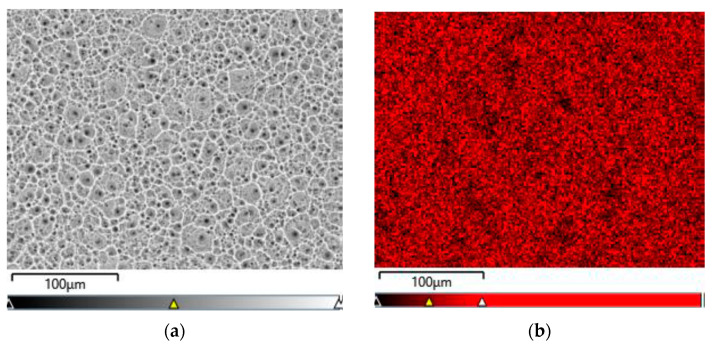

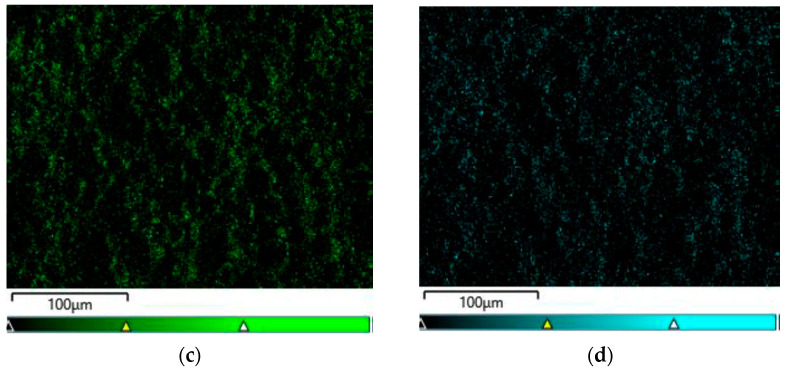
EDS analysis in the tested micro-area on the CpTi G4 surface after electrochemical oxidation at 20 V for 48 h: (**a**) FE-SEM image; (**b**) Ti surface distribution map; (**c**) O surface distribution map; (**d**) F surface distribution map.

Figure 6a–d show exemplary maps of the surface distribution of elements identified in the examined micro-area on the CpTi G4 surface after electrochemical oxidation at 20 V for 48 h, obtained using the EDS method.

The qualitative analysis of the surface chemical composition performed for all obtained porous oxide layers showed the presence of elements such as titanium, oxygen, and fluorine, and revealed their uniform distribution. The presence of oxygen results from the electrochemical oxidation process and the formation of oxide layers on the surface of CpTi G4. The EDS microanalysis also showed the presence of fluorine in the tested oxide layers, which results from the chemical composition of the electrolyte in the form of 1 M ethylene glycol with the addition of 40 g of ammonium fluoride used for oxidation. Fluoride ions originating from ammonium fluoride had the ability to be incorporated from the electrolyte into the porous oxide layers formed during the anodizing process. The incorporation of fluorine into oxide layers is a beneficial phenomenon because fluorine has bactericidal properties and can, therefore, inhibit the growth of bacteria after implantation [43]. Fluorine is a highly electronegative element, and when incorporated into biomaterials, it can alter their surface properties [44]. The presence of fluorine in oxide layers can significantly impact the biocompatibility of titanium by changing the surface energy and wettability, chemical stability, or interfacial bonding [44,45,46,47]. Fluorinated surfaces often have lower surface energy, which can reduce the wettability of the biomaterial. This can affect the adhesion and proliferation of cells on the material’s surface [45]. While reduced wettability might decrease the initial cell attachment, it can also help in preventing biofilm formation, which is beneficial for reducing the risk of implant-associated infections [46]. Fluorine-containing compounds are often chemically stable and less reactive, which can lead to better corrosion resistance and stability in biological environments [46]. This stability is crucial for long-term implants that must withstand the corrosive effects of bodily fluids. Fluorine can modify the surface chemistry of biomaterials, potentially enhancing or inhibiting the bonding between the material and the surrounding biological tissues. This can influence the integration of the material within the body, affecting its biocompatibility [47]. Fluorine can also contribute to the antibacterial properties of a biomaterial, which are crucial for preventing infections [48]. Fluorinated compounds can be used as coatings that are directly toxic to bacteria or that create a surface that bacteria find difficult to adhere to, preventing biofilm formation. Some fluorinated materials can slowly release fluoride ions, which have known antibacterial effects. Fluoride can interfere with the enzyme systems of bacteria, inhibiting their growth and metabolism. Fluorination can modify the surface charge and hydrophobicity of a biomaterial, making it less hospitable to bacterial attachment and colonization. This can be particularly effective in preventing the initial stages of biofilm formation [49]. The concentration and form of fluorine are critical. Too high a concentration can be cytotoxic to human cells as well as bacteria, affecting the material’s overall biocompatibility. There is a need to balance the benefits of antibacterial properties with potential long-term toxicity and bioaccumulation concerns. The presence of fluorine in oxide layers can significantly enhance the antibacterial properties of materials and influence their biocompatibility by modifying surface properties. However, careful design and testing are necessary to ensure that these modifications do not compromise the overall safety and effectiveness of the material in a biological context.

**Figure 7:**
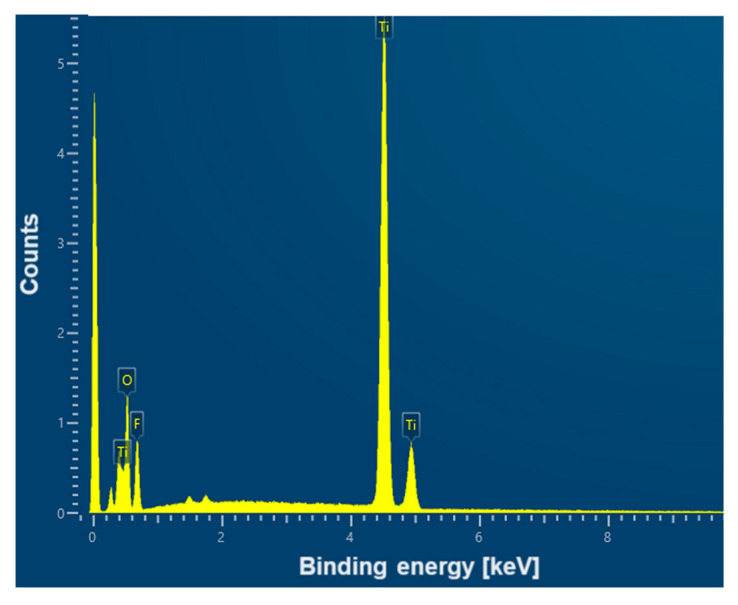
EDS spectrum from the tested micro-area of the CpTi G4 surface after electrochemical oxidation at 20 V for 48 h.

An example EDS spectrum showing the dependence of the number of counts as a function of binding energy for the CpTi G4 surface after electrochemical oxidation at 20 V for 48 h is shown in Figure 7. The EDS microanalysis confirmed the presence of chemical elements with atomic numbers Z of 22, 8, and 9, i.e., titanium, oxygen, and fluorine, respectively.

Table 1 contains the results of the quantitative microanalysis obtained on the basis of EDS spectra for the CpTi G4 surface after electrochemical oxidation at 20 V for 2 to 48 h. Local measurements of the chemical composition of the tested materials using the micro-analytical EDS method are given as the average values determined from 10 measurements for each sample along with the standard deviation in Table 1. The weight percentage of titanium, as the substrate material, and oxygen, associated with the presence of an oxide layer, is practically independent of the oxidation time used within the margin of error. The content of fluorine incorporated from the electrolyte to the surface of CpTi G4 during anodizing for a longer time of 24 and 48 h is the highest and equals 17.1 wt.%. However, it should be noted that the EDS method has limitations when determining the quantitative content of light elements, whose characteristic radiation is absorbed more intensively by the tested samples.

**Figure 8:**
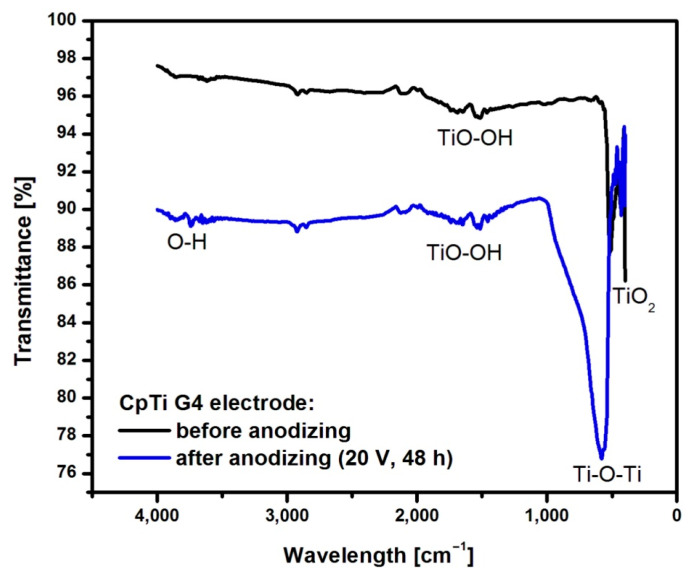
The ATR-FTIR absorption spectrum of the CpTi G4 before and after the formation of an anodic oxide layer at 20 V for 48 h.

Figure 8 shows an example ATR-FTIR spectrum for CpTi G4 before and after anodizing at 20 V for 48 h.

The spectrum obtained for CpTi G4 before anodization shows bands in the range of 400 to 500 cm^−1^ corresponding to the presence of a self-passive TiO_2_ layer in the amorphous phase [50]. The peaks at a wavelength of 1600 cm^−1^ correspond to O-O stretching vibrations inside the TiO-OH bond [51]. In the spectrum obtained for the CpTi G4 sample with a porous oxide layer, a large peak in the range of 450–1000 cm^−1^ can be observed, corresponding to the bending vibrations of the Ti-O-Ti bond, which are characteristic of an oxide layer with anatase structure [50]. At 1650 cm^−1^, stretching vibrations of the TiO-OH bond are visible. Asymmetric and symmetric stretching vibrations of the hydroxyl group (-OH) can be observed at 3400 cm^−1^ [52].

### 3.4. Surface Roughness Study

Surface roughness parameters are critical in various engineering applications, including medical device manufacturing, where they can affect the functionality, longevity, and biocompatibility of implants and instruments [53,54,55]. The most commonly used parameters include Ra (Arithmetic Average Roughness), Rz (Ten-Point Height), and Rq (Root Mean Square Roughness). Each of these parameters provides a different perspective on the texture of a surface, which can have significant implications for medical applications.

Ra is the arithmetic average of the absolute values of the profile height deviations from the mean line over the evaluation length. It is a measure of the surface’s overall roughness and is widely used due to its simplicity and ease of interpretation. A smoother surface with lower Ra can reduce the risk of bacterial adhesion and biofilm formation [53]. This is crucial for implants like hip and knee replacements, as well as for surgical instruments. For moving parts in prosthetic joints, a lower Ra value can lead to less wear and a longer lifespan of the implant due to reduced friction. Smoother surfaces can be more biocompatible, leading to better tissue integration and reduced risk of adverse reactions.

Rz is the average distance between the five highest peaks and the five lowest valleys within the sampling length. It provides information about the peak-to-valley roughness characteristics of a surface. For components subject to cyclic loading, like heart valves or dental implants, a lower Rz value can indicate better resistance to fatigue failure, as sharp peaks and valleys can act as stress concentrators [54]. In applications where lubrication is critical, such as in joint replacements, a lower Rz can help maintain a lubricating film over the surface, reducing wear. Surfaces with lower Rz values tend to be easier to clean, which is essential in preventing infections, especially in devices that come into contact with body fluids.

Rq is the square root of the sum of the squares of the individual heights and depths from the mean line. It is more sensitive to peaks and valleys than Ra and provides a more comprehensive assessment of the surface texture. Rq can be particularly useful in assessing the quality of surface finishing processes, which is critical for implants that require precise mating surfaces, such as in spinal rods and plates [55]. For load-bearing implants, a lower Rq can contribute to higher strength and durability, as it reflects a more uniform surface with fewer stress concentrators. In cases where coatings or adhesives are used, a lower Rq can lead to better adhesion due to a more uniform surface profile.

In medical applications, the choice of roughness parameter depends on the specific requirements of the device and the nature of its interaction with the biological environment. It is also important to note that while these parameters provide valuable information, they do not fully describe the complexity of a surface’s three-dimensional texture. Therefore, they are often used in conjunction with other surface characterization techniques to ensure the optimal performance and safety of medical devices.

**Figure 9:**
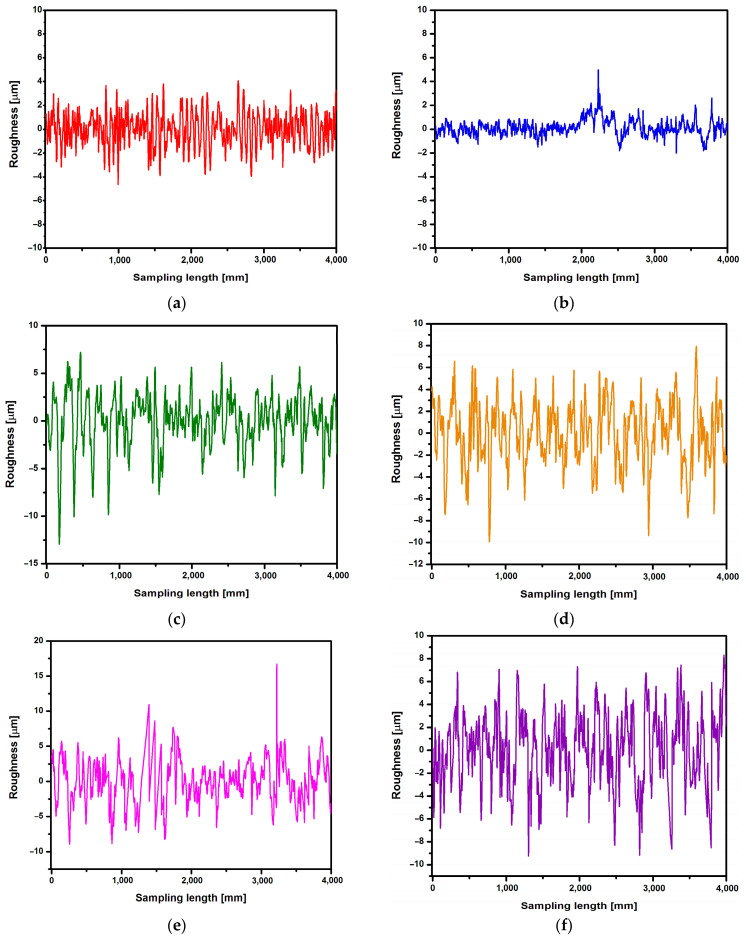
Roughness profile recorded for the CpTi G4 surface: (**a**) mechanically ground; (**b**) electrochemically polished; (**c**) anodized at 20 V for 2 h; (**d**) anodized at 20 V for 18 h; (**e**) anodized at 20 V for 24 h; and (**f**) anodized at 20 V for 48 h.

First, the roughness measurement was performed for the CpTi G4 surface before electrochemical oxidation, i.e., for the surface after mechanical grinding (Figure 9a) and after electrochemical polishing (Figure 9b). Then, roughness profiles were obtained for the CpTi G4 surface after anodizing at 20 V for 2, 18, 24, and 48 h (Figure 9c–f). Table 2 summarizes the obtained quantitative results of roughness measurements in the form of Ra, Rz, and Rq along with standard deviations for the CpTi G4 surface before and after electrochemical oxidation. Analyzing the results presented in Table 2, it can be seen that the lowest value of the Ra parameter has the electrochemically polished CpTi G4 surface, which is more than half the value of the mechanically ground surface. The obtained results confirm the fact that the electropolished surface is much smoother, which is also confirmed via microscopic observations (Figure 3a,b). The produced oxide layers are characterized by a much higher Ra parameter, as much as four or five times, compared to the surface of the CpTi G4 after electropolishing. This proves the formation of unevenness on the CpTi G4 surface in the form of a porous microstructure, the presence of which was confirmed via microscopic observations (Figure 4). It can also be noticed that the longer the electrochemical oxidation time of the CpTi G4, the higher the value of the Ra parameter, which indicates an increasingly developed porous microstructure. A similar relationship applies to the Rq parameter, with one difference being that the value of the Rq parameter increases up to an oxidation time of 24 h and maintains the same level with an oxidation time of 24 and 48 h. The value of the Rz parameter in the case of mechanical grinding is also half as high as in the case of an electropolished surface, and the highest Rz values were determined for the produced oxide layers.

The porous oxide layers produced under the proposed anodizing conditions contribute to increasing the surface roughness of CpTi G4. Moreover, the Ra parameter for the produced oxide layers takes values from the optimal range of 1 < Ra < 3, which is specified for dental implants [4].

### 3.5. Release Kinetics of Gentamicin Sulfate from Porous Oxide Layers on CpTi G4

**Figure 10:**
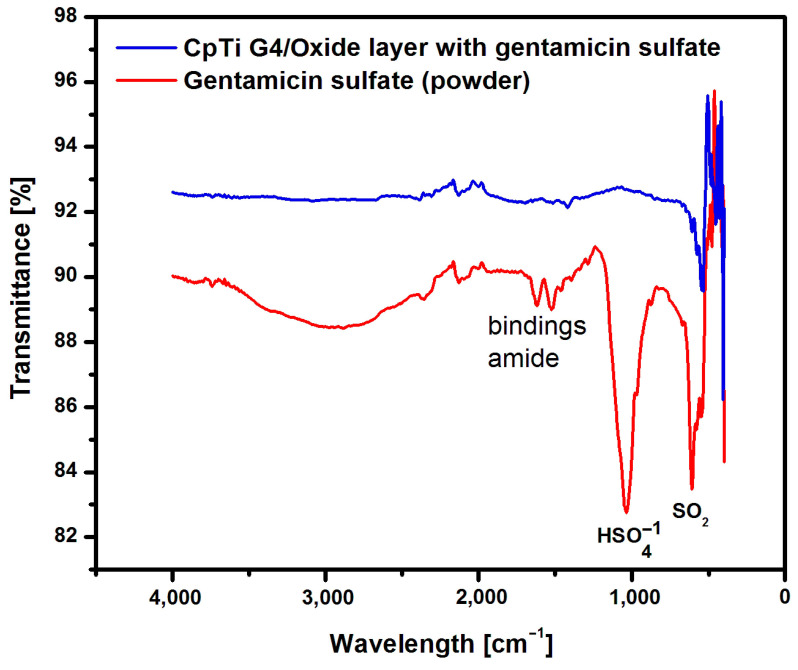
ATR-FTIR spectrum for the CpTi G4 after electrochemical oxidation at 20 V for 48 h with the drug loaded and gentamicin sulfate in the initial state.

The presence of the loaded drug into porous oxide layers on the CpTi G4 surface was confirmed with measurements carried out using the ATR-FTIR method. ATR-FTIR tests were carried out on a sample of gentamicin sulfate in powder form, i.e., in the initial state, and on the CpTi G4 sample with porous oxide layers with the drug loaded. Figure 10 shows an example ATR-FTIR spectrum for a porous oxide layer formed on the CpTi G4 surface in the electrochemical oxidation process at a voltage of 20 V for 48 h with the loaded drug and, comparatively, for gentamicin sulfate powder.

An analysis of the ATR-FTIR spectrum for gentamicin sulfate in the form of powder shown in Figure 10 showed the presence of absorption bands located at a wavelength of 1654 cm^−1^, which indicates stretching vibrations of the amide bond of the C=O group, and deformation vibrations of the amide N-H group at 1531–1453 cm^−1^ [56]. There are also visible bonds originating from the I and II amide groups present in the drug at 1029 cm^−1^ ($HSO_{4}^{-1}$ group) and 623 cm^−1^ (SO_2_ group).

The CpTi G4 sample after electrochemical oxidation with the drug loaded inside the porous microstructure of the oxide layer was also subjected to ATR-FTIR examination (Figure 10). In this case, the ATR-FTIR spectrum showed the presence of an absorption band in the range of 1650–1453 cm^−1^, which confirmed the implementation of gentamicin sulfate into the pores.

The CpTi G4 samples with porous oxide layers formed after all applied electrochemical oxidation times with loaded gentamicin sulfate were subjected to drug release kinetics examination using UV–VIS absorption spectroscopy. Table 3 shows the amount of the drug loaded into the pores depending on the anodizing time of the CpTi G4.

The data in Table 3 show that as the anodizing time increases, and, thus, the film microstructure becomes more developed, the amount of drug loaded inside the pores decreases. Based on the resulting relationship and the appearance of the microstructure in the FE-SEM microscopic images in Figure 4a–h, it can be concluded that larger and more extensive pores are not conducive to drug loading.

**Figure 11:**
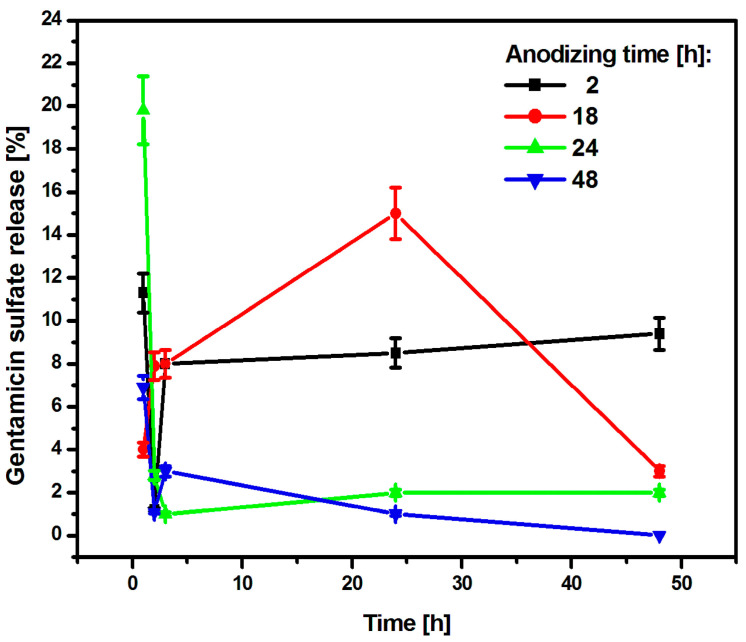
The amount of drug released in the form of gentamicin sulfate from porous oxide layers on the CpTi G4 surface as a function of release time.

The CpTi G4/Oxide layer samples after various anodizing times and loaded with the drug were subjected to a kinetics study of the release of gentamicin sulfate, which lasted 48 h. The obtained results were summarized in graphs showing the amount of the released drug as a function of the release time (Figure 11).

After an anodizing time of 2 h, the CpTi G4 sample released 11.3% of the drug substance after the first hour, and then the released amount decreased to 1.2% after the second hour. After this collapse, the amount of drug released increased again to 8%, which was recorded after the third hour. This value stabilized and remained at a similar level until the end of the study because after 24 h, the amount of drug released was 8.5%, and at the end of the study—after 48 h—it was 9.4%. It can be concluded that the substance medicinal drug was released in a regular dose. The CpTi G4 sample anodized for 18 h after the first hour released 4% of the drug and this value increased until 24 h when it amounted to 15%. Then, a gradual decrease in the amount of substance released was noted until 48 h, after which the amount of drug released was 3%. The CpTi G4 sample, after anodizing for 24 h after the first hour, showed the highest and most rapid release of the drug substance, which amounted to as much as 19.8% of the drug loaded inside the pores. In the following hours, the amount of released drug substance stabilized at approximately 2% and remained at this level until the end of the study. The CpTi G4 sample anodized for 48 h released 6.9% of the drug after the first hour and this value decreased to 1.1% after the second hour. Then, after the third hour, there was a slight increase in the amount of drug released, which amounted to 3%. After this time, the amount of drug released began to decrease again. After 24 h, it was only 1%, and at the end of the study, i.e., after 48 h, it was 0%, which means that the drug stopped being released after that time.

Comparing the obtained results of drug release kinetics, it can be concluded that the CpTi G4 sample, which was electrochemically oxidized at a voltage of 20 V for 2 h, shows the most favorable course of the drug release process because it stabilizes within the first three hours, after which the drug substance is released in a regular dose and in a controlled manner. Thanks to this, the oxide layer obtained under these anodizing conditions can be used in controlled drug delivery systems. Porous oxide layers obtained via anodizing for 24 and 48 h tend to release the drug too suddenly and too quickly, which, unfortunately, does not have a positive effect on the controlled delivery of the drug substance. For this reason, it would be necessary to use an additional layer of an appropriate polymer that would slow down the drug release and stabilize it at one level. Based on the results obtained, it can be concluded that the medicinal substance in the form of gentamicin sulfate is released from the interior of the porous oxide layers formed on the CpTi G4 surface in accordance with Fick’s first law [56], which states that the amount of substance diffusing per unit of time through the surface perpendicular to the direction is directly proportional to the area of this surface and the concentration gradient of the substance in the system.

In our earlier work [57], only 0.93 mg of gentamicin sulfate was loaded into oxide nanotubes obtained on the surface of the biomedical Ti-6Al-7Nb alloy anodized in an aqueous solution of 1 M ethylene glycol with the addition of 0.2 M NH_4_F at a voltage of 50 V for 1 h. In the case of CpTi—with the porous oxide layer obtained in the anodizing process in the electrolyte of 1M ethylene glycol with the addition of 40 g of ammonium fluoride at a voltage of 20 V for 2 h—58.6(6) mg of gentamicin sulfate was implemented inside the obtained pores. As the porosity of the oxide layer increased, the amount of the incorporated drug decreased, reaching a value of 44.2(4) mg after anodizing at 20 V for 48 h. The obtained results indicate that in the porous oxide layers on the CpTi G4 surface formed under the proposed anodizing conditions, it is possible to load larger amounts of drugs compared to the implementation of gentamicin sulfate inside the layers of oxide nanotubes on the Ti-6Al-7Nb alloy. The biomimetic oxide layers obtained in this way are promising drug carriers that ensure the prolonged release of the drug substance. The resulting pores on the surface of the anodic oxide layers create further nanopores in the already formed micropores, which ensures the greater durability of the drug delivery system.

## 4. Conclusions

Based on the results obtained, it can be concluded that electrochemical polishing improves the quality of the CpTi G4 surface by smoothing it and removing scratches and micro-cavities. The electrochemical oxidation of CpTi G4 in a solution of 1 M ethylene glycol with the addition of 40 g of ammonium fluoride at a voltage of 20 V for 2, 18, 24, and 48 h is an effective method of producing biomimetic oxide layers imitating the porous microstructure of bone tissue. Increasing the anodizing time results in the formation of more pores and a more developed microstructure of oxide layers. Anodizing contributed to increasing the surface roughness of CpTi G4, which was confirmed by the results of profilometric tests, which showed that the longer the oxidation time, the greater the surface roughness. As the anodization time of CpTi G4 increases, and, thus, the microstructure of the anodic oxide film increases, the amount of the drug in the form of gentamicin sulfate incorporated into the resulting pores decreases. It has been proven that the obtained oxide layers on the CpTi G4 surface can serve as drug carriers in targeted drug delivery systems. The most favorable release profile of gentamicin sulfate was demonstrated for CpTi G4 oxidized for 2 h. The proposed method of administering the medicinal substance released locally from the CpTi G4/Oxide layer surface allows for the omission of the oral route of antibiotic therapy.

## Figures and Tables

**Table 1:** Content of elements on the surface of CpTi G4 after electrochemical oxidation in solution at a voltage of 20 V for 2, 18, 24, and 48 h.

CpTi G4 Sample	Element [wt.%]		
	Ti	O	F
Anodized at 20 V for 2 h	54.7(6)	28.9(4)	16.4(9)
Anodized at 20 V for 18 h	54.5(2)	30.6(7)	14.9(3)
Anodized at 20 V for 24 h	54.1(6)	28.8(6)	17.1(2)
Anodized at 20 V for 48 h	53.9(4)	29.0(4)	17.1(3)

**Table 2:** Quantitative roughness measurement results for the CpTi G4 surface before and after electrochemical oxidation.

CpTi G4 Sample	Ra [µm]	Rz [µm]	Rq [µm]
Mechanically ground	1.11(19)	7.35(07)	1.38(05)
Electrochemically polished	0.50(01)	3.71(01)	0.66(02)
Anodized at 20 V for 2 h	2.08(01)	14.76(06)	2.70(05)
Anodized at 20 V for 18 h	2.27(01)	13.99(02)	2.80(01)
Anodized at 20 V for 24 h	2.49(08)	16.92(02)	3.12(09)
Anodized at 20 V for 48 h	2.54(05)	15.32(01)	3.11(08)

**Table 3:** The amount of drug loaded in the form of gentamicin sulfate into the porous oxide layers on the CpTi G4 surface.

Anodizing Time [h]	Amount of Gentamicin Sulfate Loaded [mg]
2	58.6(6)
12	51.9(5)
24	46.3(4)
48	44.2(4)

## Data Availability

The data presented in this study are available on request from the corresponding author.
